# A discrete-time analog for coupled within-host and between-host dynamics in environmentally driven infectious disease

**DOI:** 10.1186/s13662-018-1522-1

**Published:** 2018-02-26

**Authors:** Buyu Wen, Jianpeng Wang, Zhidong Teng

**Affiliations:** College of Mathematics and Systems Science, Xinjiang University, Urumqi, People’s Republic of China

**Keywords:** 92D30, 39A60, Within-host dynamics, Between-host dynamics, NSFD scheme, Threshold value, Stability

## Abstract

In this paper, we establish a discrete-time analog for coupled within-host and between-host systems for an environmentally driven infectious disease with fast and slow two time scales by using the non-standard finite difference scheme. The system is divided into a fast time system and a slow time system by using the idea of limit equations. For the fast system, the positivity and boundedness of the solutions, the basic reproduction number and the existence for infection-free and unique virus infectious equilibria are obtained, and the threshold conditions on the local stability of equilibria are established. In the slow system, except for the positivity and boundedness of the solutions, the existence for disease-free, unique endemic and two endemic equilibria are obtained, and the sufficient conditions on the local stability for disease-free and unique endemic equilibria are established. To return to the coupling system, the local stability for the virus- and disease-free equilibrium, and virus infectious but disease-free equilibrium is established. The numerical examples show that an endemic equilibrium is locally asymptotically stable and the other one is unstable when there are two endemic equilibria.

## Introduction

As is well known, viruses have caused abundant types of epidemic and occur almost everywhere on Earth, infecting humans, animals, plants, and so on. There are a large number of diseases, for example: influenza, hepatitis, HIV, AIDS, SARS, Ebola, MERS, etc., which are caused by viruses. Therefore, it is important to study viral infection, which can supply theory evidence for controlling diseases breaking out.

In recent years, many authors have established and investigated the various kinds of viral infection dynamical systems which are described by differential equations and difference equations. Many important and valuable results were established and successfully applied to viral infections in practice. See, for example, [1–22] and the references therein.

In [1–4], the authors proposed a coupled within-host and between-host continuous-time dynamical system:

1 $$ \textstyle\begin{cases} \frac{\mathrm{d}S}{\mathrm{d}t} = A-\beta ES-\mu S, \\ \frac{\mathrm{d}I}{\mathrm{d}t} = \beta ES-(\mu+\alpha) I, \\ \frac{\mathrm{d}E}{\mathrm{d}t} = \theta IV(1-E)-\gamma E, \\ \frac{\mathrm{d}T}{\mathrm{d}t} =\Lambda-kVT-mT, \\ \frac{\mathrm{d}T^{*}}{\mathrm{d}t} =kVT-(m+d)T^{*}, \\ \frac{\mathrm{d}V}{\mathrm{d}t} =g(E)+pT^{*}-cV, \end{cases} $$

where *S*, *I*, *E*, *T*, $T^{*}$ and *V* denote the numbers of susceptible and infectious individuals, the level of environment contamination, the densities of healthy cells and infected cells, and the parasite load, respectively. In system (1), the parameter *A* and Λ denote the recruitment rate of susceptible and healthy cells, respectively. *β* is the infection rate of hosts in a contaminated environment. *μ* is the natural mortality rate of host. *θ* is the rate of contamination. *γ* is the clearance rate. *k* is the infection rate of cells. *m* and *d* denote the natural and infection-induced mortality rates of infected cells, respectively. *p* is the parasite reproduction rate by an infected cell. *c* is the within-host mortality rate of parasites. *α* is the induced mortality rate of host. It is assumed that the rate of environment contamination is proportional to the number of infected hosts and the parasite load *V* within a host, which has the form $\theta VI$. The function $g(E)$ denotes the rate at which an average host is inoculated.

In [1], for system (1) the authors introduced a slow time variable $\tau=\varepsilon t$, where $0<\varepsilon\ll1$. In this case, *t* as a fast time variable. The authors further considered the parameters associated with the dynamics at the population level to be small based on the assumption that the between-host dynamics occur on a slower time scale than that of parasite-cell dynamics within the host. Let $\Lambda=\varepsilon \tilde{\Lambda}$, $\beta=\varepsilon\tilde{\beta}$, $\mu =\varepsilon\tilde{\mu}$, $\alpha=\varepsilon\tilde{\alpha}$, $\theta=\varepsilon\tilde{\theta}$ and $\gamma=\varepsilon\tilde {\gamma}$. Then, under the faster time variable *t* and the slower time variable *τ*, system (1) can be written as the two singular perturbation systems, see systems (6) and (7) given in [1], respectively. Using the techniques from the singular perturbation theory in [23], the authors in [1] analyzed system (1) by analyzing the corresponding fast and slow dynamics, and the fast and slow dynamics can be analyzed using the fast and slow time subsystems, see systems (8) and (9) given in [1], respectively. Here, we see that the concepts of fast and slow time systems were introduced in [23] early.

We easily see that system (1) also can be described by using fast and slow time variables *t* and *τ* in the following form:

2 $$ \textstyle\begin{cases} \frac{\mathrm{d}S}{\mathrm{d}\tau} = A-\beta ES-\mu S, \\ \frac{\mathrm{d}I}{\mathrm{d}\tau} = \beta ES-(\mu+\alpha) I, \\ \frac{\mathrm{d}E}{\mathrm{d}\tau} = \theta IV(1-E)-\gamma E, \\ \frac{\mathrm{d}T}{\mathrm{d}t} =\Lambda-kVT-mT, \\ \frac{\mathrm{d}T^{*}}{\mathrm{d}t} =kVT-(m+d)T^{*}, \\ \frac{\mathrm{d}V}{\mathrm{d}t} =g(E)+pT^{*}-cV. \end{cases} $$

From this system, by the transformations $t=\frac{\tau}{\varepsilon }$ for the last three equations of system (2) and $\tau =\varepsilon t$ for the first three equations of system (2), we can easily obtain two similar singular perturbation systems and two similar fast and slow time subsystems as systems (6), (7) and systems (8), (9) proposed in [1].

In recent years, more and more attention was paid on the discrete-time epidemic models. The reasons are as follows. Firstly, because the statistic data about infectious disease is collected by day, week, month, or year, so it is more direct, more convenient and more accurate to describe the epidemic by using discrete-time models than continuous-time models. Secondly, it is very difficult to solve a nonlinear differential equation with a given initial condition to obtain the exact solution. Thus, for many practical requirements, such as numerical calculation, it is often necessary to discretize a continuous model into the corresponding discrete model. Therefore, we see that the discrete-time analog is also alike important for studying coupled system (1). At the present time, there are various discretization methods to discretize a continuous model, including the standard methods, such as the Euler method, the Runge–Kutta method, and some other standard finite difference schemes, and the non-standard finite difference scheme, which is originally developed by Mickens (see [24–26]).

In this paper, we propose a discrete-time analog for above continuous-time system (2) by using discretization method of Micken’s non-standard finite difference scheme, the model is given as follows:

3 $$ \textstyle\begin{cases} S(t+1)-S(t) = A-\beta E(t)S(t+1)-\mu S(t+1), \\ I(t+1)-I(t) = \beta E(t)S(t+1)-(\mu+\alpha) I(t+1), \\ E(t+1)-E(t) = \theta I(t)V(s+1)(1-E(t+1))-\gamma E(t+1), \end{cases} $$

and

4 $$ \textstyle\begin{cases} T(s+1)-T(s) = \Lambda-kV(s)T(s+1)-mT(s+1), \\ T^{*}(s+1)-T^{*}(s) = kV(s)T(s+1)-(m+d)T^{*}(s+1), \\ V(s+1)-V(s) = g(E(t+1))+pT^{*}(s+1)-cV(s+1), \end{cases} $$

where system (3) denotes the slow dynamics with slow time *t*, and system (4) denotes the fast dynamics with fast time *s*. However, in slow system (3) there is a fast time term $V(s+1)$, and in fast system (4) there is a slow time term $g(E(t+1))$. Therefore, systems (3) and (4) form a coupled system.

It is clear that in order to study the dynamical properties of coupled systems (3)–(4) we can firstly analyze the fast and slow two subsystems which are determined by two time scales *t* and *s*. In other words, we can treat the within-host subsystem (4) as the fast system and the between-host subsystem (3) as the slow system.

For fast time *s* and slow time *t*, we may assume that there exists a certain relation between *s* and *t*. For example, it may be assumed that there is a large enough integer *K* such that $s=Kt$ in slow system (3) and $t=[\frac{s}{K}]$ in fast system (4), where $[\frac {s}{K}]$ denotes the maximum integer which is not more than $\frac {s}{K}$. Thus, coupled systems (3)–(4) will acquire the following form:

5 $$ \textstyle\begin{cases} S(t+1)-S(t) = A-\beta E(t)S(t+1)-\mu S(t+1), \\ I(t+1)-I(t) = \beta E(t)S(t+1)-(\mu+\alpha) I(t+1), \\ E(t+1)-E(t) = \theta I(t)V(Kt+1)(1-E(t+1))-\gamma E(t+1), \\ T(s+1)-T(s) = \Lambda-kV(s)T(s+1)-mT(s+1), \\ T^{*}(s+1)-T^{*}(s) = kV(s)T(s+1)-(m+d)T^{*}(s+1), \\ V(s+1)-V(s) = g(E([\frac{s}{K}]+1))+pT^{*}(s+1)-cV(s+1). \end{cases} $$

However, since there are terms $V(Kt+1)$ and $g(E([\frac{s}{K}]+1))$ in system (5), it is very difficult to readily investigate system (5) theoretically.

Therefore, in this paper we firstly separate the coupled systems (3)–(4) into a fast system and a slow system by using the idea of limit systems. For fast system (4), we assume that the environmental contamination *E* keeps a constant owing to the faster time scale is enough quick. For slow system (3), we can assume that $V(s)$ steadies to an equilibrium $\hat{V}(E)$. Thus, coupled systems (3)–(4) are separated into the following two isolated subsystems:

6 $$ \textstyle\begin{cases} T(s+1)-T(s) = \Lambda-kV(s)T(s+1)-mT(s+1), \\ T^{*}(s+1)-T^{*}(s) = kV(s)T(s+1)-(m+d)T^{*}(s+1), \\ V(s+1)-V(s) = g(E)+pT^{*}(s+1)-cV(s+1), \end{cases} $$

and

7 $$ \textstyle\begin{cases} S(t+1)-S(t) =A-\beta E(t)S(t+1)-\mu S(t+1), \\ I(t+1)-I(t) =\beta E(t)S(t+1)-(\mu+\alpha) I(t+1), \\ E(t+1)-E(t) =\theta I(t)\hat{V}(E(t+1))(1-E(t+1))-\gamma E(t+1), \end{cases} $$

where $\hat{V}(E)$ is given in Sect. 3.

In this paper, for fast system (6) we will investigate the dynamical behaviors, including the positivity, boundedness, basic reproduction number, the existence of equilibria and the local stability of equilibria by using the discretization method. For slow system (7), we will investigate the dynamical properties, including the positivity, boundedness, the existence of disease-free equilibrium, only a unique endemic equilibrium, and two endemic equilibria, and the local asymptotic stability for the disease-free and endemic equilibria.

Next, we will investigate the dynamical behaviors for the coupled systems (3)–(4) basing on the research results obtained for the fast and slow subsystems. We will establish some criteria on the local asymptotic stability for the infection- and disease-free equilibrium, virus infectious but disease-free equilibrium and the endemic equilibrium. Furthermore, for the special cases which there is a unique endemic equilibrium, and two endemic equilibria in coupled systems (3)–(4), by means of the numerical examples, we will indicate that the unique endemic equilibrium may be locally asymptotically stable, and an endemic equilibrium may be locally asymptotically stable but the other one may be unstable.

This paper is organized as follows. In Sects. 2 and 3, fast system (6) and slow system (7) are discussed. Some criteria on the positivity, boundedness, existence of equilibria and local asymptotic stability are stated and proved. In Sect. 4, coupled systems (3)–(4) is discussed. Some criteria on the existence of equilibria and local asymptotic stability are stated and proved. In Sect. 5, the numerical examples are given. Lastly, a discussion is presented in Sect. 6.

## The analysis of fast system

We firstly introduce the following lemmas on the quadratic and cubic polynomial equations which are given in [27].

### Lemma 1

*All roots* *λ* *of the quadratic equation* $f(\lambda)=\lambda ^{2}-A\lambda+B=0$ *satisfy* $|\lambda|<1$ *if and only if the following conditions are satisfied*:

$$B< 1, \qquad f(-1)=1+A+B>0,\qquad f(1)=1-A+B>0. $$

### Lemma 2

*All roots* *λ* *of the cubic equation* $f(\lambda)=\lambda ^{3}+a_{2}\lambda^{2}+a_{1}\lambda+a_{0}=0$ *satisfy* $|\lambda|<1$ *if and only if the following conditions are satisfied*:

$$f(1)>0,\qquad (-1)^{3}f(-1)>0,\qquad |a_{0}|< 1,\qquad |b_{0}|>|b_{2}|, $$

*where* $b_{0}=a^{2}_{0}-1$ *and* $b_{2}=a_{0}a_{2}-a_{1}$.

For coupled systems (3)–(4), function $g(E)$ is assumed to satisfy the following basic assumption.

(H) $g(E)$ is defined for all $0 \leq E\leq1$ and is continuously differentiable, which satisfies $g(0)=0$, $g(E)\geq0$, $g'(E)>0$ and $g''(E)\leq0$ for all $0 \leq E\leq1$.

From the biological background of system (6), it is assumed that any solution $(T(s),T^{*}(s), V(s))$ satisfies the following initial value:

8 $$ T(0)>0, \qquad T^{*}(0)>0, \qquad V(0)>0. $$

Firstly, on the positivity and boundedness of the solutions and the existence of nonnegative equilibria for system (6) we have the following results.

### Lemma 3

*The solution* $(T(s),T^{*}(s),V(s))$ *of system* (6) *with initial value* (8) *is positive for all* $s\geq0$ *and ultimately bounded*.

### Proof

System (6) is equivalent to the following:

9 $$ \textstyle\begin{cases} T(s+1)= \frac{\Lambda+T(s)}{1+m+kV(s)}, \\ T^{*}(s+1)= \frac{T^{*}(s)}{1+m+d}+\frac{kV(s)(\Lambda +T(s))}{(1+m+d)(1+m+kV(s))}, \\ V(s+1)= \frac{g(E)}{1+c}+\frac{V(s)}{1+c}+\frac {pT^{*}(s)}{(1+c)(1+m+d)} \\ \hphantom{V(s+1)={}}{} +\frac{pkV(s)(\Lambda+T(s))}{(1+c)(1+m+d)(1+m+kV(s))}. \end{cases} $$

If (8) is satisfied, then from system (9) it follows that $(T(1),T^{*}(1),V(1))$ exists and is positive. Hence, by induction, we see that $(T(s),T^{*}(s),V(s))$ exists and is positive for all $s\geq0$.

From the first equation of system (9), we have

$$T(s+1)\leq\frac{\Lambda}{1+m}+\frac{1}{1+m}T(s). $$

Hence,

10 $$ \limsup_{s\rightarrow\infty}T(s)\leq\frac{\Lambda}{m} \triangleq T_{0}. $$

Particularly, when $T(0)\leq T_{0}$, we also have $T(s)\leq T_{0}$ for all $s>0$.

From the second equation of system (9), we have

$$T^{*}(s+1)\leq\frac{T^{*}(s)}{1+m+d}+\frac{\Lambda+T(s)}{1+m+d}. $$

By (10), we can obtain

11 $$ \limsup_{s\rightarrow\infty} T^{*}(s)\leq\bar{T}^{*} \triangleq\frac {m+1}{m+d}T_{0}. $$

Particularly, we also can prove that when $T(0)\leq T_{0}$ and $T^{*}(0)\leq\bar{T}^{*}$, then $T^{*}(s)\leq\bar{T}^{*}$ for all $s>0$.

From the third equation of system (9), it follows that

$$V(s+1)\leq\frac{g(E)}{1+c}+\frac{V(s)}{1+c}+\frac{pT^{*}(s)}{(1+c)(1+m+d)} + \frac{p(\Lambda+T(s))}{(1+c)(1+m+d)}. $$

By (10) and (11), we can obtain

$$\limsup_{s \rightarrow\infty} V(s)\leq\bar{V}\triangleq\frac {g(E)m(1+m+d)(m+d)+2p\Lambda(m+1)}{cm(1+m+d)(m+d)}. $$

Similarly, we also can prove that $V(s)\leq\bar{V}$ for all $s>0$ if $T(0)\leq T_{0}$, $T^{*}(0)\leq\bar{T}^{*}$ and $V(0)\leq\bar{V}$.

Therefore, solution $(T(s),T^{*}(s),V(s))$ with initial value (8) is ultimately bounded. This completes the proof. □

### Remark 1

Define a set as follows:

$$\Gamma=\bigl\{ \bigl(T,T^{*},V\bigr): 0\leq T\leq T_{0}, 0\leq T^{*}\leq \bar{T}^{*}, 0\leq V\leq\bar{V}\bigr\} . $$

Then from the proof of Lemma 3 we see that Γ is a positive invariable and globally attractive set for system (6).

We define the baseline within-host reproduction number as follows:

$$R_{f}=\frac{kpT_{0}}{c(m+d)}. $$

### Lemma 4

*Let* $E=0$, *then system* (6) *always has infection*-*free equilibrium* $B_{0}(T_{0},0,0)$, *and when* $R_{f}>1$, *system* (6) *has a unique infectious equilibrium* $B^{*}(\breve{T},\breve{T}^{*},\breve{V})$.

### Proof

Let $E=0$. It is obvious that system (5) has a unique infection-free equilibrium $B_{0}(T_{0},0,0)$. For infectious equilibrium $B^{*}(\breve {T},\breve{T}^{*},\breve{V})$, we have

$$ \textstyle\begin{cases} \Lambda-k\breve{V}\breve{T}-m\breve{T}=0, \\ k\breve{V}\breve{T}-(m+d)\breve{T}^{*}=0, \\ p\breve{T}^{*}-c\breve{V}^{*}=0. \end{cases} $$

Hence,

$$\breve{T}^{*}=\frac{cm}{pk}(R_{f}-1), \qquad \breve{T}=\frac {(m+d)c}{kp},\qquad \breve{V}=\frac{pmT_{0}}{c(m+d)} \biggl(1- \frac{1}{R_{f}} \biggr). $$

This shows that, when $R_{f}>1$, an infectious equilibrium $B^{*}(\breve {T},\breve{T}^{*},\breve{V})$ exists and is unique. This completes the proof. □

Let $E>0$ in system (6). If $(\breve{T}(E),\breve{T}^{*}(E),\breve {V}(E))$ is a nonnegative equilibrium of system (6), then we have

12 $$ \textstyle\begin{cases} \Lambda-k\breve{V}(E)\breve{T}(E)-m\breve{T}(E)=0, \\ k\breve{V}(E)\breve{T}(E)-(m+d)\breve{T}^{*}(E)=0, \\ g(E)+p\breve{T}^{*}(E)-c\breve{V}(E)=0. \end{cases} $$

We further have

$$\breve{V}(E)=\frac{1}{c}\bigl(g(E)+p\breve{T}^{*}(E)\bigr), \qquad \breve {T}^{*}(E)=\frac{m}{m+d}\bigl(T_{0}- \breve{T}(E)\bigr) $$

and $\breve{T}(E)$ satisfies the following equation:

13 $$ \breve{T}^{2}(E)-a_{1}\breve{T}(E)+a_{2}=0, $$

where

$$a_{1}=\frac{g(E)(m+d)}{pm}+T_{0} \biggl(1+\frac{1}{R_{f}} \biggr)>0,\qquad a_{2}=\frac{T^{2}_{0}}{R_{f}}>0. $$

Since

$$\begin{aligned} a^{2}_{1}-4a_{2}&= \biggl[\frac{g(E)(m+d)}{pm}+T_{0} \biggl(1+\frac {1}{R_{f}}\biggr) \biggr]^{2}-\frac{4T^{2}_{0}}{R_{f}} \\ &> T^{2}_{0} \biggl(1+\frac{1}{R_{f}} \biggr)^{2}-\frac {4T^{2}_{0}}{R_{f}}\geq0, \end{aligned}$$

Eq. (13) has always two positive real solutions given by the following:

14 $$ \breve{T}_{\pm}(E)=\frac{1}{2} \Bigl(a_{1}\pm\sqrt {a^{2}_{1}-4a_{2}} \Bigr). $$

Note that $a'_{1}(E)=g'(E)\frac{m+d}{pm}>0$, and

15 $$ \breve{T}'_{\pm}(E)=\frac{1}{2}a'_{1}(E) \biggl(1\pm\frac {a_{1}}{\sqrt{a^{2}_{1}-4a_{2}}} \biggr). $$

Owing to $a_{2}>0$, we have from (15) for any $E\geq0$, $\breve {T}'_{+}(E)>0$ and $\breve{T}'_{-}(E)<0$.

In (14), when $E=0$, by calculating we obtain

$$\breve{T}_{\pm}(0)=\frac{1}{2} \biggl[T_{0}\biggl(1+ \frac{1}{R_{f}}\biggr)\pm\sqrt {T^{2}_{0}\biggl(1- \frac{1}{R_{f}}\biggr)^{2}} \biggr]. $$

Therefore,

16 $$ \breve{T}_{+}(0)= \textstyle\begin{cases} T_{0}, &\mbox{if } R_{f}\geq1, \\ \frac{T_{0}}{R_{f}}, &\mbox{if } R_{f}< 1, \end{cases} $$

and

17 $$ \breve{T}_{-}(0)= \textstyle\begin{cases} \frac{T_{0}}{R_{f}}, &\mbox{if } R_{f} > 1, \\ T_{0}, &\mbox{if } R_{f}\leq1. \end{cases} $$

Since $\breve{T}'_{+}(E)>0$ and $\breve{T}_{+}(0)\geq T_{0}$ from (16), we obtain $\breve{T}_{+}(E)>T_{0}$ for all $E>0$. But, from the first equation of (12), we have $\breve{T}_{+}(E) \leq T_{0}$. This leads to a contradiction. Since $\breve{T}'_{-}(E)<0$ and $\breve {T}_{-}(0)\leq T_{0}$ from (17), we obtain $\breve{T}_{-}(E)<\breve {T}_{-}(0)\leq T_{0}$ for all $E\geq0$. Therefore, when $E>0$, system (6) has a unique positive equilibrium $B_{1}(\breve{T}(E),\breve {T}^{*}(E),\breve{V}(E))$, where

$$\breve{T}(E)=\breve{T}_{-}(E),\qquad \breve{T}^{*}(E)= \frac{m}{m+d}\bigl(T_{0}-\breve{T}(E)\bigr) $$

and

$$\breve{V}(E)= \frac{1}{c} \biggl[g(E)+\frac{mp}{m+d} \bigl(T_{0}-\breve {T}(E)\bigr) \biggr]. $$

By calculating, we also have

$$\breve{T}(E)=\frac{T_{0}}{R_{v}(E)},\qquad \breve{T}^{*}(E)= \frac{\Lambda}{m+d} \biggl(1-\frac{1}{R_{v}(E)} \biggr) $$

and

$$\breve{V}(E)= \frac{1}{c} \biggl[g(E)+\frac{p\Lambda}{m+d} \biggl(1- \frac {1}{R_{v}(E)} \biggr) \biggr], $$

where

$$R_{v}(E)=\frac{2T_{0}}{a_{1}-\sqrt{a^{2}_{1}-4a_{2}}}. $$

Furthermore, when $E\to0^{+}$, from (14) and (17), by calculating we can obtain

$$B_{1}\bigl(\breve{T}(E),\breve{T}^{*}(E),\breve{V}(E)\bigr) \to \textstyle\begin{cases} B_{0}(T_{0},0,0), &\mbox{if } R_{f}\leq1, \\ B^{*}(\breve{T},\breve{T}^{*},\breve{V}), &\mbox{if } R_{f}>1. \end{cases} $$

Summarizing the above discussions, we finally get the following result.

### Lemma 5

*Let* $E>0$, *then fast system* (6) *always has a unique infected equilibrium* $B_{1}(\breve{T}(E),\breve{T}^{*}(E),\breve{V}(E))$, *and*

$$\lim_{E\to0^{+}}B_{1}\bigl(\breve{T}(E), \breve{T}^{*}(E),\breve {V}(E)\bigr)= \textstyle\begin{cases} B_{0}(T_{0},0,0), &\textit{if } R_{f}\leq1, \\ B^{*}(\breve{T},\breve{T}^{*},\breve{V}), &\textit{if } R_{f}>1. \end{cases} $$

Next, we discuss the stability of the infection-free equilibrium and infectious equilibrium for system (6). We have the following theorems.

### Theorem 1

*Let* $E=0$ *in system* (6).

- *If* $R_{f}<1$, *then infection*-*free equilibrium* $B_{0}$ *is locally asymptotically stable*.
- *If* $R_{f}>1$, *then* $B_{0}$ *is unstable*.

### Proof

The linearization system of system (6) at equilibrium $B_{0}$ is

$$ \textstyle\begin{cases} X(s+1) =-kT_{0}Z(s)-mX(s+1)+X(s), \\ Y(s+1) =kT_{0}Z(s)-(m+d)Y(s+1)+Y(s), \\ Z(s+1) =pY(s+1)-cZ(s+1)+Z(s), \end{cases} $$

which is equivalent to the following:

18 $$ \textstyle\begin{cases} X(s+1) =\frac{1}{1+m}X(s)-\frac{kT_{0}}{1+m}Z(s), \\ Y(s+1) =\frac{1}{1+m+d}Y(s)+\frac{kT_{0}}{1+m+d}Z(s), \\ Z(s+1) =\frac{p}{(1+c)(1+m+d)}Y(s)+\frac{1}{1+c} (\frac {pkT_{0}}{1+m+d}+1 )Z(s). \end{cases} $$

The characteristic equation of system (18) is

$$\varphi(\lambda)= \biggl(\lambda-\frac{1}{1+m} \biggr)f(\lambda)= 0, $$

where

$$f(\lambda)=\lambda^{2}- \biggl(\frac{1}{1+c} +\frac{pkT_{0}}{(1+m+d)(1+c)}+ \frac{1}{1+m+d} \biggr)\lambda+\frac {1}{(1+d+m)(1+c)}. $$

We have eigenvalues $\lambda_{1}=\frac{1}{1+m}$, and $\lambda_{2}$ and $\lambda_{3}$ satisfying the equation $f(\lambda)=0$. It is clear that $f(0)<1$, $f(-1)>0$ and

$$\begin{aligned} \begin{aligned} f(1)& = 1- \biggl(\frac{1}{1+c}+\frac{pkT_{0}}{(1+m+d)(1+c)} +\frac{1}{1+m+d} \biggr)+\frac{1}{(1+d+m)(1+c)} \\ &= \frac{c(d+m)-pkT_{0}}{(1+d+m)(1+c)}. \end{aligned} \end{aligned}$$

When $R_{f}<1$, then $f(1)>0$. By Lemma 1, it follows $|\lambda_{2}|<1$ and $|\lambda_{3}|<1$. Therefore, equilibrium $B_{0}$ is locally asymptotically stable.

When $R_{f}>1$, then $f(1)<0$. Since $\lim_{\lambda\to\infty }f(\lambda)=+\infty$, we see that $f(\lambda)=0$ has a root $\lambda _{3}\in(1,+\infty)$. This implies that equilibrium $B_{0}$ is unstable. This completes the proof. □

### Theorem 2

*Let* $E=0$ *in system* (6). *If* $R_{f}>1$, *then infectious equilibrium* $B^{*}$ *is locally asymptotically stable*.

The proof of Theorem 2 will be given in Theorem 3 as the special case with $E=0$. We hence omit it here.

### Theorem 3

*Let* $E>0$ *in system* (6). *Then infectious equilibrium* $B_{1}(\breve {T}(E),\breve{T}^{*}(E), \breve{V}(E))$ *is locally asymptotically stable*.

### Proof

We will prove Theorem 3 in the case $E\geq0$, and when $E=0$ we assume that $R_{f}>1$ and $B_{1}(\breve{T}(E),\breve{T}^{*}(E),\breve {V}(E))=B^{*}(\breve{T},\breve{T}^{*},\breve{V})$. We first prove for all $E\geq0$

19 $$ \breve{T}(E)\leq\frac{c(m+d)}{pk}. $$

In fact, from the third equation of (12) and $g(E)\geq0$, we have $c\breve{V}(E)\geq p\breve{T}^{*}(E)$. From the second equation of (12), then $\breve{V}(E)=\frac{(m+d)\breve{T}^{*}(E)}{k\breve{T}(E)}$. This shows that

$$\frac{c(m+d)\breve{T}^{*}(E)}{k\breve{T}(E)}\geq p\breve{T}^{*}(E). $$

Therefore, $\breve{T}(E)\leq\frac{c(m+d)}{pk}$.

For convenience, let $\breve{T}(E)=\breve{T}$, $\breve {T}^{*}(E)=\breve{T}^{*}$ and $\breve{V}(E)=\breve{V}$. The linearization system of system (6) at equilibrium $B_{1}$ is

20 $$ \textstyle\begin{cases} X(s+1) = X(s)-k\breve{V}X(s+1)-k\breve{T}Z(s)-mX(s+1), \\ Y(s+1) = k \breve{T}Z(s)+k\breve{V}X(s+1)-(m+d)Y(s+1)+Y(s), \\ Z(s+1) = pY(s+1)-cZ(s+1)+Z(s). \end{cases} $$

The characteristic equation of system (20) is

$$g(\lambda)= \lambda^{3}+a_{1}\lambda^{2}+a_{2} \lambda+a_{3}= 0, $$

where

$$\begin{aligned}& a_{1}= - \biggl[\frac{1}{1+c}+\frac{1}{1+m+d}+ \frac{pk\breve {T}(1+m)}{(1+c)(1+m+d)(1+m+k\breve{V})} +\frac{1}{1+m+k\breve{V}} \biggr], \\& a_{2}= \frac{1}{(1+m+d)(1+c)}+\frac{1}{(1+m+k\breve{V})(1+c)} +\frac{1}{(1+m+d)(1+m+k\breve{V})} \\& \hphantom{a_{2}={}}{} +\frac{pk\breve{T}(1+m)}{(1+c)(1+m+d)(1+m+k\breve{V})^{2}} +\frac{pk\breve{V}k\breve{T}}{(1+c)(1+m+d)(1+m+k\breve{V})^{2}}, \\& a_{3}= -\frac{1}{(1+m+k\breve{V})(1+m+d)(1+c)}. \end{aligned}$$

Obviously, $|a_{3}|<1$. According to (19), we obtain

$$g(1)= \frac{c(m+d)(m+k\breve{V})-pkm\breve {T}}{(1+c)(1+m+d)(1+m+k\breve{V})}>0. $$

Further, we have

$$\begin{aligned} (-1)^{3}g(-1) =& 1+\frac{1}{1+c}+\frac{1}{1+m+d}+ \frac{pk\breve {T}(1+m)}{(1+c)(1+m+d)(1+m+k\breve{V})} \\ &{} +\frac{1}{1+m+k\breve{V}}+\frac{1}{(1+m+d)(1+c)}+\frac {1}{(1+m+k\breve{V})(1+c)} \\ &{} +\frac{1}{(1+m+d)(1+m+k\breve{V})} +\frac{pk\breve{T}(1+m)}{(1+c)(1+m+d)(1+m+k\breve{V})^{2}} \\ &{} +\frac{k\breve{T}pk\breve{V}}{(1+c)(1+m+d)(1+m+k\breve {V})^{2}} \\ &{} +\frac{1}{(1+m+k\breve{V})(1+m+d)(1+c)}> 0. \end{aligned}$$

Now, we prove $|b_{0}|+b_{2}>0$ and $|b_{0}|-b_{2}>0$, where $b_{0}=1-a_{3}^{2}$ and $b_{2}=a_{1}a_{3}-a_{2}$. Since

$$\begin{aligned}& |b_{0}| = 1-\frac{1}{(1+m+k\breve{V})^{2}(1+m+d)^{2}(1+c)^{2}}, \\& b_{2} = \frac{1}{(1+m+k\breve{V})(1+m+d)(1+c)} \biggl[\frac {1}{1+c}+ \frac{1}{1+m+d} \\& \hphantom{b_{2} ={}}{}+\frac{pk\breve{T}(1+m)}{(1+c)(1+m+d)(1+m+k\breve{V})}+\frac {1}{1+m+k\breve{V}} \biggr] \\& \hphantom{b_{2} ={}}{}-\frac{1}{(1+m+k\breve{V})} \biggl[\frac{1}{1+c} + \frac{1}{1+m+d} +\frac{pk\breve{T}(1+m)}{(1+c)(1+m+d)(1+m+k\breve{V})} \biggr] \\& \hphantom{b_{2} ={}}{}-\frac {1}{(1+m+d)(1+c)} -\frac{k\breve{T}pk\breve{V}}{(1+c)(1+m+d)(1+m+k\breve{V})^{2}}, \end{aligned}$$

we have

$$\begin{aligned} |b_{0}|-b_{2} =&1-\frac{1}{(1+m+k\breve{V})^{2}(1+m+d)^{2}(1+c)^{2}} \\ &{} +\frac{1}{(1+m+d)(1+c)} \biggl[1-\frac{1}{(1+m+k\breve {V})^{2}} \biggr] \\ &{} +\frac{1}{1+m+k\breve{V}} \biggl[\frac{1}{1+c}+\frac {1}{1+m+d} \biggr] \biggl[1-\frac{1}{(1+m+d)(1+c)} \biggr] \\ &{} +\frac{pk\breve{T}(1+m)}{(1+c)(1+m+d)(1+m+k\breve{V})^{2}} \biggl[1-\frac{1}{(1+m+d)(1+c)} \biggr] \\ &{} +\frac{k\breve{T}pk\breve{V}}{(1+c)(1+m+d)(1+m+k\breve{V})^{2}}>0, \end{aligned}$$

and according to (19), we further have

$$\begin{aligned} |b_{0}|+b_{2} \geq&\frac{1}{(1+m+k\breve {V})^{2}(1+m+d)^{2}(1+c)^{2}} \bigl[ \bigl(c+m+d+c(m+d)\bigr) \\ &{} \times(1+m+d) (1+c) (1+m+k\breve{V})^{2}+\bigl(c+m+d+c(m+d)\bigr) \\ &{} -\bigl(c+m+d+c(m+d)\bigr) (2+m+d+c) (1+m+k\breve{V}) \\ &{} -c(m+d) \bigl(m+c+d+c(m+d)\bigr) (1+m+k\breve{V})-c(m+d)k\breve{V} \bigr] \\ =& \frac{1}{(1+m+k\breve{V})^{2}(1+m+d)^{2}(1+c)^{2}} \bigl[\bigl(c+m+d+c(m+d)\bigr) \\ &{} \times(1+c) (1+m+k\breve{V}) (m+k\breve {V})-\bigl(c+m+d+c(m+d)\bigr) (m+k \breve{V}) \\ &{} +\bigl(c+m+d+c(m+d)\bigr) (m+d) (1+c) (1+m+k\breve{V}) (m+k\breve{V}) \\ &{} -c(m+d)k\breve{V} \bigr]> 0. \end{aligned}$$

Therefore, by Lemma 2 all roots *λ* of the equation $g(\lambda )=0$ satisfy $|\lambda|<1$. Thus, equilibrium $B_{1}$ is local asymptotically stable. Particularly, when $E=0$ we also see that equilibrium $B^{*}$ is local asymptotically stable. This completes the proof. □

## The analysis of slow system

Now, we consider slow system (7). We assume that fast system (6) has steadied at the equilibrium $\hat{B}(\hat{T}(E),\hat {T}^{*}(E),\hat{V}(E))$, where $\hat{B}(\hat{T}(E),\hat {T}^{*}(E),\hat{V}(E))$ is defined as follows:

$$\hat{B}\bigl(\hat{T}(E),\hat{T}^{*}(E),\hat{V}(E)\bigr)= \textstyle\begin{cases} B_{1}(\breve{T}(E),\breve{T}^{*}(E),\breve{V}(E)),& \mbox{if } E>0, \\ B^{*}(\breve{T},\breve{T}^{*},\breve{V}),& \mbox{if } E=0, R_{f}>1, \\ B_{0}(T_{0},0,0),& \mbox{if } E=0, R_{f}\leq1. \end{cases} $$

It is clear that fast system (6) is locally asymptotically stable in equilibrium *B̂*, that is, when $E>0$ then by Theorem 3 $B_{1}(\breve{T}(E),\breve{T}^{*}(E),\breve{V}(E))$ is local asymptotically stable, and when $E=0$ by Theorems 1 and 2 if $R_{f}>1$ then $B^{*}(\breve{T},\breve{T}^{*},\breve{V})$ is local asymptotically stable and if $R_{f}<1$ then $B_{0}(T_{0},0,0)$ is local asymptotically stable. Therefore, we can choose $V(t+1)=\hat {V}(E(t+1))$ in slow system (7).

From the biological background of system (7), we assume that any solution $(S(t),I(t), E(t))$ of system (7) satisfies the initial value

21 $$ S(0)>0, \qquad I(0)\geq0, \qquad 0\leq E(0)\leq1. $$

Firstly, on the positivity and boundedness of the solutions and the existence of nonnegative equilibria for slow system (7) we have the following lemmas.

### Lemma 6

*The solution* $(S(t),I(t),E(t))$ *of system* (7) *with initial value* (21) *is positive for all* $t\geq0$ *and ultimately bounded*. *Furthermore*, $0\leq E(t)\leq1$ *for all* $t\geq0$.

### Proof

We know that system (7) is equivalent to the following form:

22 $$ \textstyle\begin{cases} S(t+1) =\frac{A+S(t)}{1+\mu+\beta E(t)}, \\ I(t+1) =\frac{\beta E(t)S(t+1)+I(t)}{1+\mu+\alpha}, \\ E(t+1) =\frac{\theta I(t)\hat{V}(E(t+1))+E(t)}{1+\gamma+\theta I(t)\hat{V}(E(t+1))}. \end{cases} $$

When $t=0$, we prove that $(S(1), I(1), E(1))$ is positive. In fact, according to the first equation of system (22), we have $S(1)=\frac{A+S(0)}{1+\mu+\beta E(0)}>0$. Next, according to the second equations of system (22) and $S(1)>0$, we have $I(1)=\frac{\beta E(0)S(1)+I(0)}{1+\mu+\alpha}>0$. Furthermore, according to the third equation of system (22), we have

$$E(1)=\frac{\theta I(0)\hat{V}(E(1))+E(0)}{1+\gamma+\theta I(0)\hat {V}(E(1))}. $$

Let $x=E(1)$, then the above equality becomes $f(x)=0$, where

$$f(x)=(1+\gamma)x+\theta I(0) \hat{V}(x) (x-1)-E(0). $$

We have $f(0)=-\theta I(0)\hat{V}(0)-E(0)<0$ and $f(1)=1+\gamma-E(0)>0$. Hence, $f(x)=0$ has at least one solution. We have

$$f''(x)=2\theta I(0)\hat{V}'(x)+\theta I(0) (x-1)\hat{V}''(x). $$

When $E>0$ we have

$$\hat{V}(E)=\breve{V}(E)=\frac{1}{c} \biggl[g(E)+\frac {mp}{m+d} \bigl(T_{0}-\breve{T}(E)\bigr) \biggr]. $$

Hence,

$$\hat{V}'(x)=\frac{1}{c} \biggl[g'(x)- \frac{mp}{m+d}\breve{T}'(x) \biggr],\qquad \hat{V}''(x)= \frac{1}{c} \biggl[g''(x)-\frac{mp}{m+d} \breve {T}''(x) \biggr]. $$

According to assumption (H), (15) and $\breve{T}(x)= \breve {T}_{-}(x)$, we know that $\hat{V}'(x)>0$ and

$$\breve{T}''(x)=\frac{1}{2}a''_{1}(x) \biggl(1-\frac{a_{1}}{\sqrt {a^{2}_{1}-4a_{2}}} \biggr) -\frac{1}{2}\bigl(a'_{1}(x) \bigr)^{2}\frac{1-\frac {a^{2}_{1}}{a^{2}_{1}-4a_{2}}}{\sqrt{a^{2}_{1}-4a_{2}}}, $$

where $a''_{1}(x)=\frac{m+d}{pm}g''(x)<0$. This shows $\breve{T}''(x)>0$, which leads to $\hat{V}''(x)<0$. Therefore, $f''(x)>0$, this implies that $f'(x)=0$ has at most one solution. Hence, there is a unique $x>0$ such that $f(x)=0$. Therefore, $(S(1),I(1),E(1))$ exists uniquely and is positive. Furthermore, from $0\leq E(0)\leq1$ we also have $0< E(1)<1$.

When $t=1$, by a similar argument to above, we can prove that $(S(2),I(2),E(2))$ exists uniquely and is positive. Owing to $0< E(1)<1$, we also have $0< E(2)<1$. Using induction, for any $t\geq0$, we know that $(S(t),I(t),E(t))$ exists uniquely and is positive. Furthermore, we finally have $0< E(t)<1$ for all $t\geq0$.

Now, we prove that $(S(t),I(t),E(t))$ is ultimately bounded. From the first equation of system (22), we have

$$S(t+1)\leq\frac{A}{1+\mu}+\frac{1}{1+\mu}S(t). $$

Hence, $\limsup_{t\rightarrow\infty}S(t)\leq\frac{A}{\mu}$. From the second equation of system (22), we have

$$I(t+1)\leq\frac{\beta A}{\mu(1+\mu+\alpha)}+\frac{1}{1+\mu +\alpha}I(t). $$

It follows that $\limsup_{t\rightarrow\infty}I(t)\leq\frac{\beta A}{\mu(\mu +\alpha)}$. This completes the proof. □

Noticing the slow system (7) and the quick system (6) is linked by the terms $V(s)$ and $g(E)$. Then if $R_{f}>1$, there are the steadied infectious equilibrium in the quick system (6). In this case, we give the basic reproduction number $R_{s}$ for slow system (7):

$$R_{s}=\frac{pmT_{0}}{c(m+d)} \biggl(1-\frac{1}{R_{f}} \biggr) \frac{\theta \beta A}{\gamma\mu(\mu+\alpha)}. $$

Obviously, we see that if $R_{f}<1$ then $R_{s}<0$, if $R_{f}=1$ then $R_{s}=0$ and if $R_{f}>1$ then $R_{s}>0$. Furthermore, when $R_{s}\geq1$ then we must have $R_{f}>1$. We denote the functions as follows:

$$F(E)=(1-E)\hat{V}(E),\qquad G(E)=\frac{\gamma(\mu+\alpha) E}{\theta A}+\frac{\gamma(\mu+\alpha)\mu}{\theta A\beta} $$

and

$$H(E)=F(E)-G(E),\qquad H_{M}=\max_{0\leq E\leq1}\bigl\{ H(E) \bigr\} . $$

Based on the reproduction number $R_{s}$, we have the following lemma.

### Lemma 7

(i) *System* (7) *always has a disease*-*free equilibrium* $P_{0}(\frac {A}{\mu},0,0)$.
(ii) *System* (7) *has a unique endemic equilibrium* $P^{*}(\bar{S},\bar {I},\bar{E})$ *if and only if one of the following conditions holds*:
  - $R_{s}=1$ *and* $H_{M}>0$;
  - $R_{s}>1$.
(iii) *System* (7) *has two endemic equilibria* $P_{1}(S_{1},I_{1},E_{1})$ *and* $P_{2}(S_{2},I_{2},E_{2})$ *if and only if the following condition holds*:
  (c) $R_{s}<1$ *and* $H_{M}>0$.
(iv) *System* (7) *has only disease*-*free equilibrium* $P_{0}(\frac{A}{\mu },0,0)$ *if and only if one of the following conditions holds*:
  (d) $H_{M}<0$;
  (e) $R_{s}=1$ *and* $H_{M}=0$.

### Proof

It is obvious that system (7) has disease-free equilibrium $P_{0}(\frac {A}{\mu},0,0)$. The endemic equilibrium $P^{*}(\bar{S},\bar{I},\bar {E})$ satisfies equation

$$ \textstyle\begin{cases} A-\beta\bar{E}\bar{S}-\mu\bar{S}=0, \\ \beta\bar{E}\bar{S}-(\mu+\alpha)\bar{I}=0, \\ \theta\bar{I}\hat{V}(\bar{E})(1-\bar{E})-\gamma\bar{E}=0. \end{cases} $$

Hence, we have

$$\bar{S}=\frac{A}{\beta\bar{E}+\mu}, \qquad \bar{I}=\frac{A\beta \bar{E}}{(\mu+\alpha)(\beta\bar{E}+\mu)} $$

and

$$(1-\bar{E})\hat{V}(\bar{E}) =\frac{\gamma(\mu+\alpha)\bar{E}}{\theta A}+\frac{\gamma\mu(\mu +\alpha)}{\theta A\beta}, $$

which is equivalent to $H(\bar{E})=0$. By calculating, we have

$$H(0)= \textstyle\begin{cases} {-}\frac{\gamma(\mu+\alpha)\mu}{\theta A\beta}, &\mbox{if } R_{f}\leq1, \\ \frac{p\Lambda}{c(m+d)} (1-\frac{1}{R_{f}} )-\frac{\gamma (\mu+\alpha)\mu}{\theta A\beta}, &\mbox{if } R_{f}>1, \end{cases} $$

and $H(1)=-\frac{\gamma(\mu+\alpha)}{\theta A}-\frac{\gamma(\mu +\alpha)\mu}{\theta A\beta}<0$. It is clear that when $R_{s}<1$ then $H(0)<0$, when $R_{s}>1$ then $H(0)>0$ and when $R_{s}=1$ then $H(0)=0$.

Furthermore, by calculating we see that, when $0< E\leq1$,

$$H''(E)= -\frac{2}{c} \biggl[g'(E)- \frac{mp}{m+d}\breve{T}'(E) \biggr] +\frac{1-E}{c} \biggl[g''(E)-\frac{mp}{m+d}\breve{T}''(E) \biggr]. $$

Hence, $H''(E)<0$ for all $0< E\leq1$. This shows that $H(E)$ is as above a convex function.

If condition (a) holds, then from $H(0)=0$ and $H_{M}>0$, we easily see that $H(E)=0$ has a unique positive root *Ē*. Hence, endemic equilibrium $P^{*}(\bar{S},\bar{I},\bar{E})$ exists and is unique.

If condition (b) holds, then from $H(0)>0$, it follows that $H(E)=0$ has a unique positive root *Ē*, and hence endemic equilibrium $P^{*}(\bar{S},\bar{I},\bar{E})$ also exists and is unique.

Assume that condition (c) holds, then owing to $H(0)<0$ and $H_{M}>0$, $H(E)=0$ has only two positive roots. Hence, system (7) has only two endemic equilibria $P_{1}$ and $P_{2}$.

Lastly, we prove that system (7) has only disease-free equilibrium $P_{0}(\frac{A}{\mu},0,0)$ if one of the conditions (d) and (e) holds. In fact, when $H_{M}<0$ we see that $H(E)=0$ has no root. When $R_{s}=1$, then $H(0)=0$. Therefore, by $H_{M}=0$ there is only disease-free equilibrium $P_{0}(\frac{A}{\mu},0,0)$. This completes the proof. □

### Remark 2

From the proof of Lemma 7, we further see that when system (7) has a unique endemic equilibrium $P^{*}(\bar{S},\bar{I},\bar{E})$, then since $H(E)$ is a above convex function for $0\leq E\leq1$, we also have $H'(\bar{E})\leq0$.

Next, we discuss the stability of the disease-free equilibrium and endemic equilibrium for system (7). We have the following theorems.

### Theorem 4

- *If* $R_{s}\leq1$, *then disease*-*free equilibrium* $P_{0}$ *of system* (7) *is locally asymptotically stable*.
- *If* $R_{s}>1$ *then equilibrium* $P_{0}$ *is unstable*.

### Proof

The linearization part of system (22) is

23 $$ \textstyle\begin{cases} X(t+1) =\frac{1}{1+\mu}X(t)-\frac{\beta A}{\mu(1+\mu)}Z(t), \\ Y(t+1) =\frac{1}{1+\mu+\alpha}Y(t)+\frac{\beta A}{\mu(1+\mu +\alpha)}Z(t), \\ Z(t+1) =\frac{\theta\hat{V}(0)}{1+\gamma}Y(t)+\frac{1}{1+\gamma}Z(t). \end{cases} $$

The characteristic equation of system (23) is

$$p(\lambda)= \biggl(\lambda-\frac{1}{1+\mu} \biggr)f(\lambda)=0, $$

where

$$f(\lambda)=\lambda^{2}- \biggl(\frac{1}{1+\mu+\alpha}+\frac {1}{1+\gamma} \biggr)\lambda+\frac{\mu-\beta\theta A\hat{V}(0)}{\mu (1+\mu+\alpha)(1+\gamma)}. $$

When $R_{f}\leq1$, we have $\hat{V}(0)=0$. Hence $f(\lambda)=0$ has roots $\lambda_{1}=\frac{1}{1+\mu+\alpha}$ and $\lambda_{2}=\frac {1}{1+\gamma}$. This implies that disease-free equilibrium $E^{0}$ is locally asymptotically stable.

When $R_{f}>1$, we have $\hat{V}(0)=V^{*}$. Owing to $R_{s}\leq1$, by calculating we obtain

$$\begin{aligned}& f(0) =\frac{1-R_{s}\gamma(\mu+\alpha)}{(1+\mu+\alpha)(1+\gamma )}< 1, \\& f(1) =1-\frac{1+\gamma+1+\mu+\alpha}{(1+\mu+\alpha)(1+\gamma)} +\frac{1-R_{s}\gamma(\mu+\alpha)}{(1+\mu+\alpha)(1+\gamma)} \\& \hphantom{f(1)}>\frac{(1+\mu+\alpha)(1+\gamma)-(1+\gamma)-(1+\mu+\alpha)+1 -\gamma(\mu+\alpha)}{(1+\mu+\alpha)(1+\gamma)}=0, \\& f(-1) =1+\frac{1+\gamma+1+\mu+\alpha}{(1+\mu+\alpha)(1+\gamma)} +\frac{1-R_{s}\gamma(\mu+\alpha)}{(1+\mu+\alpha)(1+\gamma)}>0. \end{aligned}$$

We see that by Lemma 1 two roots $\lambda_{1}$ and $\lambda_{2}$ of $f(\lambda)=0$ satisfy $|\lambda_{1}|<1$ and $|\lambda_{2}|<1$. Therefore, disease-free equilibrium $P_{0}$ is locally asymptotically stable.

When $R_{s}>1$, we have

$$\begin{aligned} f(1)&=1-\frac{1+\gamma+1+\mu+\alpha}{(1+\mu+\alpha)(1+\gamma)} +\frac{1-R_{s}\gamma(\mu+\alpha)}{(1+\mu+\alpha)(1+\gamma)} \\ &< \frac{(1+\mu+\alpha)(1+\gamma)-(1+\gamma)-(1+\mu+\alpha)+1 -\gamma(\mu+\alpha)}{(1+\mu+\alpha)(1+\gamma)}=0 \end{aligned}$$

and $\lim_{\lambda\to\infty}f(\lambda)=\infty$, there is a $\bar {\lambda}>1$ such that $f(\bar{\lambda})=0$. Therefore, $P_{0}$ is unstable. □

In order to discuss the stability of unique endemic equilibrium $P^{*}(\bar{S},\bar{I},\bar{E})$ of system (7), we need to introduce the following assumption.

(A) $(\varphi+1)(n+1)(w-1)M+(1+2r+\varphi)N>0$, where $M=\varphi w+\varphi n+nw+nw\varphi+r(1+ \mu)\varphi-2$, $N=w\varphi+n\varphi+nw+2(\varphi+n+w)+2-r\varphi$, $\varphi=\mu+\alpha$, $w=\gamma-\theta\bar{I}F'(\bar{E})$ and $n=\mu+\beta\bar{E}$.

### Theorem 5

*Assume that* (A) *holds and one of conditions* (a) *and* (b) *in Lemma *7 *holds*. *Then unique endemic equilibrium* $P^{*}(\bar{S},\bar{I},\bar{E})$ *of system* (7) *is locally asymptotically stable*.

### Proof

The linearization part of system (22) is

24 $$ \textstyle\begin{cases} X(t+1) =\frac{1}{1+\mu+\beta\bar{E}}X(t)-\frac{\beta\bar {S}}{1+\mu+\beta\bar{E}}Z(t), \\ Y(t+1) =\frac{\beta\bar{E}}{(1+\mu+\alpha)(1+\mu+\beta\bar {E})}X(t)+\frac{1}{1+\mu+\alpha}Y(t) \\ \hphantom{Y(t+1) ={}}{}+\frac{(1+\mu)\beta\bar{S}}{(1+\mu+\alpha)(1+\mu+\beta\bar {E})}Z(t), \\ Z(t+1) =\frac{\theta\hat{V}(\bar{E})(1-\bar{E})}{1+\gamma-\theta \bar{I}F'(\bar{E})}Y(t)+\frac{1}{1+\gamma-\theta\bar{I}F'(\bar{E})}Z(t). \end{cases} $$

Here, by calculating we have

$$1+\gamma-\theta\bar{I}F'(\bar{E})= 1+\gamma-\frac{\gamma\beta\bar{E}}{\beta\bar{E}+\mu}- \theta \bar{I}H'(\bar{E})>1. $$

The characteristic equation of system (24) is

$$f(\lambda)= \lambda^{3}+a_{1}\lambda^{2}+a_{2} \lambda+a_{3}= 0, $$

where

$$\begin{aligned}& a_{1} = -\biggl[\frac{1}{1+\mu+\beta\bar{E}}+\frac{1}{1+\mu+\alpha }+ \frac{1}{1+\gamma-\theta\bar{I}F'(\bar{E})}\biggr], \\& a_{2} =\frac{(1+\mu)(1-\gamma(\mu+\alpha))+\beta\bar{E}+(1+\mu +\alpha)+(1+\gamma-\theta\bar{I}F'(\bar{E}))}{ (1+\mu+\alpha)(1+\mu+\beta\bar{E})(1+\gamma-\theta\bar {I}F'(\bar{E}))}, \\& a_{3} = -\frac{1-\gamma(\mu+\alpha)}{(1+\mu+\alpha)(1+\mu+\beta \bar{E})(1+\gamma-\theta\bar{I}F'(\bar{E}))}. \end{aligned}$$

Obviously, $|a_{3}|<1$. By calculating, we can obtain

$$f(1)=\frac{(\mu+\alpha)(\mu+\beta\bar{E})(\gamma-\theta\bar {I}F'(\bar{E})) -\gamma\mu(\mu+\alpha)}{(1+\mu+\alpha)(1+\mu+\beta\bar{E}) (1+\gamma-\theta\bar{I}F'(\bar{E}))}>0. $$

Further, we have

$$\begin{aligned} (-1)^{3}f(-1) =& 1+ \biggl[\frac{1}{1+\mu+\beta\bar{E}}+\frac{1}{1+\mu+\alpha }+ \frac{1}{1+\gamma-\theta\bar{I}F'(\bar{E})} \biggr] \\ &{} +\frac{(1+\mu)(1-\gamma(\mu+\alpha))+\beta\bar{E}+(1+\mu +\alpha)+(1+\gamma-\theta\bar{I}F'(\bar{E}))}{ (1+\mu+\alpha)(1+\mu+\beta\bar{E})(1+\gamma-\theta\bar {I}F'(\bar{E}))} \\ &{} +\frac{1-\gamma(\mu+\alpha)}{(1+\mu+\alpha)(1+\mu+\beta \bar{E})(1+\gamma-\theta\bar{I}F'(\bar{E}))}> 0. \end{aligned}$$

Now, we prove $|b_{0}|+b_{2}>0$ and $|b_{0}|-b_{2}>0$, where $b_{0}=1-a_{3}^{2}$ and $b_{2}=a_{1}a_{3}-a_{2}$. Since

$$\begin{aligned}& |b_{0}|= 1-\frac{(1-\gamma(\mu+\alpha))^{2}}{(1+\mu+\alpha )^{2}(1+\mu+\beta\bar{E})^{2}(1+\gamma-\theta\bar{I}F'(\bar {E}))^{2}}, \\& b_{2}= \frac{1-\gamma(\mu+\alpha)}{(1+\mu+\alpha)(1+\mu+\beta \bar{E})(1+\gamma-\theta\bar{I}F'(\bar{E}))} \biggl[\frac{1}{1+\mu+\beta\bar{E}}+ \frac{1}{1+\mu+\alpha} \\& \hphantom{b_{2}={}}{}+\frac{1}{1+\gamma-\theta\bar{I}F'(\bar{E})} \biggr] -\frac{(1+\mu)(1-\gamma(\mu+\alpha))}{(1+\mu+\alpha)(1+\mu +\beta\bar{E})(1+\gamma-\theta\bar{I}F'(\bar{E}))} \\& \hphantom{b_{2}={}}{} -\frac{\beta\bar{E}+(1+\mu+\alpha)+(1+\gamma-\theta\bar {I}F'(\bar{E}))}{ (1+\mu+\alpha)(1+\mu+\beta\bar{E})(1+\gamma-\theta\bar {I}F'(\bar{E}))}, \end{aligned}$$

by calculating we can obtain

$$\begin{aligned} |b_{0}|-b_{2} =&1-\frac{(1-\gamma(\mu+\alpha))^{2}}{(1+\mu+\alpha)^{2}(1+\mu +\beta\bar{E})^{2} (1+\gamma-\theta\bar{I}F'(\bar{E}))^{2}} \\ &{} +\frac{1}{(1+\mu+\beta\bar{E})(1+\gamma-\theta\bar {I}F'(\bar{E}))} \biggl[1-\frac{1-\gamma(\mu+\alpha)}{(1+\mu+\alpha)^{2}} \biggr] \\ &{} +\frac{1}{(1+\mu+\beta\bar{E})(1+\mu+\alpha)} \biggl[1-\frac{1-\gamma(\mu+\alpha)}{(1+\gamma-\theta\bar {I}F'(\bar{E}))^{2}} \biggr] \\ &{} +\frac{[(1+\mu)(1-\gamma(\mu+\alpha))+\beta\bar{E}][1+\mu +\beta\bar{E}]-1+\gamma(\mu+\alpha)}{ (1+\mu+\alpha)(1+\mu+\beta\bar{E})^{2}(1+\gamma-\theta\bar {I}F'(\bar{E}))}>0 \end{aligned}$$

and

$$\begin{aligned} |b_{0}|+b_{2} =&Q^{-1} \bigl[(1+\mu+ \alpha)^{2}(1+\mu+\beta\bar {E})^{2}\bigl(1+\gamma-\theta \bar{I}F'(\bar{E})\bigr)^{2} \\ &{} -\bigl(1-\gamma(\mu+\alpha)\bigr)^{2}+\bigl(1-\gamma(\mu+\alpha) \bigr)\bigl[(1+\mu +\alpha) \bigl(1+\gamma-\theta\bar{I}F'(\bar{E}) \bigr) \\ &{} +(1+\mu+\beta\bar{E}) \bigl(1+\gamma-\theta\bar{I}F'(\bar {E}) \bigr)+(1+\mu+\alpha) (1+\mu+\beta\bar{E})\bigr] \\ &{} -(1+\mu) \bigl(1-\gamma(\mu+\alpha)\bigr) (1+\mu+\alpha) (1+\mu+\beta \bar{E}) \bigl(1+\gamma-\theta\bar{I}F'(\bar{E})\bigr) \\ &{} -\beta\bar{E}(1+\mu+\alpha) (1+\mu+\beta\bar{E}) \bigl(1+\gamma -\theta \bar{I}F'(\bar{E})\bigr) \\ &{} -(1+\mu+\alpha)^{2}(1+\mu+\beta\bar{E}) \bigl(1+\gamma-\theta \bar{I}F'(\bar{E})\bigr) \\ &{} -(1+\mu+\alpha) (1+\mu+\beta\bar{E}) \bigl(1+\gamma-\theta\bar {I}F'(\bar{E})\bigr)^{2} \bigr] \\ =& Q^{-1} \bigl[(1+\mu+\alpha)^{2}(1+\mu+\beta \bar{E})^{2}\bigl(1+\gamma -\theta\bar{I}F'(\bar{E}) \bigr)^{2} \\ &{} -\bigl(1-\gamma(\mu+\alpha)\bigr)^{2}+\bigl(1-\gamma(\mu+\alpha) \bigr)\bigl[(1+\mu +\alpha) \bigl(1+\gamma-\theta\bar{I}F'(\bar{E}) \bigr) \\ &{} +(1+\mu+\beta\bar{E}) \bigl(1+\gamma-\theta\bar{I}F'(\bar {E}) \bigr)+(1+\mu+\alpha) (1+\mu+\beta\bar{E})\bigr] \\ &{} +\gamma(1+\mu) (\mu+\alpha) (1+\mu+\alpha) (1+\mu+\beta\bar {E}) \bigl(1+ \gamma-\theta\bar{I}F'(\bar{E})\bigr) \\ &{} -(1+\mu+\alpha) (1+\mu+\beta\bar{E})^{2}\bigl(1+\gamma-\theta \bar{I}F'(\bar{E})\bigr) \\ &{} -(1+\mu+\alpha)^{2}(1+\mu+\beta\bar{E}) \bigl(1+\gamma-\theta \bar{I}F'(\bar{E})\bigr) \\ &{} -(1+\mu+\alpha) (1+\mu+\beta\bar{E}) \bigl(1+\gamma-\theta\bar {I}F'(\bar{E})\bigr)^{2} \bigr] \\ =&Q^{-1} \bigl[(\varphi+1) (n+1) (w-1)M+(1+2r+\varphi)N \bigr], \end{aligned}$$

where

$$Q=(1+\mu+\alpha)^{2}(1+\mu+\beta\bar{E})^{2}\bigl(1+\gamma- \theta\bar {I}F'(\bar{E})\bigr)^{2}=(1+ \varphi)^{2}(1+n)^{2}(1+w)^{2}. $$

From assumption (A) we obtain $|b_{0}|+b_{2}>0$. By Lemma 2, all roots *λ* of the equation $f(\lambda)=0$ satisfy $|\lambda |<1$. Therefore, equilibrium $P^{*}$ locally asymptotically stable. □

It is difficult to discuss the local stability for the case of two positive equilibria $P_{1}$ and $P_{2}$ in condition (c) of Lemma 7 by using the linearization method. However, we can give the following conjecture.

### Conjecture 1

*Assume that* $R_{s}<1$ *and* $H_{M}>0$. *Let* $P_{1}(\bar{S}_{1},\bar{I}_{1},\bar {E}_{1})$ *and* $P_{2}(\bar{S}_{2},\bar{I}_{2},\bar{E}_{2})$ *be two positive equilibria of slow system* (7) *with* $\bar{E}_{1}<\bar{E}_{2}$. *Then* $P_{2}$ *is locally asymptotically stable*, *and* $P_{1}$ *is unstable*.

## The analysis for coupled system

Now, we return to coupled systems (3)–(4). If $\tilde{D}(\tilde {S},\tilde{I},\tilde{E},\tilde{T},\tilde{T^{*}},\tilde{V})$ is the equilibrium of coupled systems (3)–(4), then we have

$$ \textstyle\begin{cases} A-\beta\tilde{E}\tilde{S}-\mu\tilde{S}=0, \\ \beta\tilde{E}\tilde{S}-(\mu+\alpha)\tilde{I}=0, \\ \theta\tilde{I}\tilde{V}(1-\tilde{E})-\gamma\tilde{E}=0, \\ \Lambda-k\tilde{V}\tilde{T}-m\tilde{T}=0, \\ k\tilde{V}\tilde{T}-(m+d)\tilde{T}^{*}=0, \\ g(\tilde{E})+p\tilde{T}^{*}-c\tilde{V}=0. \end{cases} $$

From Lemmas 4, 5 and 7, we have the following result.

### Lemma 8

1. *Coupled system* (3)*–*(4) *always has a disease*-*free and infection*-*free equilibrium* $D_{0}(\frac{A}{\mu},0,0,T_{0},0,0)$.
2. *If* $R_{f}>1$, *then coupled system* (3)*–*(4) *has a disease*-*free equilibrium* $D_{1}(\frac{A}{\mu},0,0, \breve{T},\breve {T}^{*},\breve{V})$.
3. *If one of the conditions* (a) *and* (b) *in Lemma *7 *holds*, *then coupled system* (3)*–*(4) *has a unique endemic equilibrium* $D^{*}(\bar{S},\bar{I},\bar{E},\breve{T}(\bar{E}),\breve {T}^{*}(\bar{E}),\breve{V}(\bar{E}))$.
4. *If the condition* (d) *in Lemma *7 *holds*, *then coupled system* (3)*–*(4) *has only two positive equilibria* $D_{2}(\bar{S_{1}},\bar{I_{1}},\bar{E_{1}},\breve{T}(\bar {E_{1}}),\breve{T}^{*}(\bar{E_{1}}),\breve{V}(\bar{E_{1}}))$ *and* $D_{3}(\bar{S_{2}},\bar{I_{2}},\bar{E_{2}},\breve{T}(\bar{E_{2}}), \breve{T}^{*}(\bar{E_{2}}), \breve{V}(\bar{E_{2}}))$ *with* $\bar {E_{1}}<\bar{E_{2}}$.

On the stability of equilibrium $\tilde{D}(\tilde{S},\tilde {I},\tilde{E},\tilde{T},\tilde{T}^{*},\tilde{V})$ of coupled system (3) and (4), we have the following definition.

### Definition 1

1. *D̃* is said to be stable, if for any constant $\epsilon >0$, there is a $\delta=\delta(\epsilon)>0$ such that, for any initial point $(S_{0},I_{0},E_{0},T_{0},T^{*}_{0},V_{0})$ at time $s=0$ and $t=0$ satisfying $|S_{0}-\tilde{S}|<\delta$, $|I_{0}-\tilde {I}|<\delta$, $|E_{0}-\tilde{E}|<\delta$, $|T_{0}-\tilde{T}|<\delta$, $|T^{*}_{0}-\tilde{T^{*}}|<\delta$, and $|V_{0}-\tilde{V}|<\delta$, one has $|S(t)-\tilde{S}|<\delta$, $|I(t)-\tilde{I}|<\delta$, $|E(t)-\tilde{E}|<\delta$, $|T(s)-\tilde{T}|<\delta$, $|T^{*}(s)-\tilde{T^{*}}|<\delta$, and $|V(s)-\tilde{V}|<\delta$, for all $t\geq0$ and $s\geq0$.
2. *D̃* is said to be locally asymptotically stable, if *D̃* is stable and there is a constant $\delta>0$ such that, for any solution $(S(t),I(t),E(t),T(s),T^{*}(s),V(s))$ with initial point $(S_{0},I_{0},E_{0},T_{0},T^{*}_{0},V_{0})$ at time $s=0$ and $t=0$ satisfying $|S_{0}-\tilde{S}|<\delta$, $|I_{0}-\tilde {I}|<\delta$, $|E_{0}-\tilde{E}|<\delta$, $|T_{0}-\tilde{T}|<\delta$, $|T^{*}_{0}-\tilde{T^{*}}|<\delta$, and $|V_{0}-\tilde{V}|<\delta$, one has
  $$\lim_{t\rightarrow\infty}\bigl(S(t),I(t),E(t)\bigr)=(\tilde{S},\tilde {I}, \tilde{E}),\qquad \lim_{s\rightarrow\infty}\bigl(T(s),T^{*}(s),V(s) \bigr)=\bigl(\tilde{T},\tilde {T^{*}},\tilde{V}\bigr). $$

Furthermore, by applying the theory of limit equations, from Theorems 1, 2 and 4, we have the following result.

### Theorem 6

1. *If* $R_{f}<1$ *and* $R_{s}\leq1$, *then equilibrium* $D_{0}(\frac{A}{\mu },0,0,T_{0},0,0)$ *is locally asymptotically stable*, *and if* $R_{f}>1$, *then* $D_{0}$ *is unstable*.
2. *If* $R_{f}>1$ *and* $R_{s}\leq1$, *then equilibrium* $D_{1}(\frac{A}{\mu },0,0,\breve{T},\breve{T}^{*},\breve{V})$ *is locally asymptotically stable*, *and if* $R_{s}>1$, *then* $D_{1}$ *is unstable*.

### Proof

In fact, the linearization systems of coupled systems (3)–(4) at equilibrium $D_{0}$ is

25 $$ \textstyle\begin{cases} X(t+1) =\frac{1}{1+\mu}X(t)-\frac{\beta A}{\mu(1+\mu)}Z(t), \\ Y(t+1) =\frac{1}{1+\mu+\alpha}Y(t)+\frac{\beta A}{\mu(1+\mu +\alpha)}Z(t), \\ Z(t+1) =\frac{1}{1+\gamma}Z(t), \\ U(s+1) =\frac{1}{1+m}U(s)-\frac{kT_{0}}{1+m}W(s), \\ V(s+1) =\frac{1}{1+m+d}V(s)+\frac{kT_{0}}{1+m+d}W(s), \\ W(s+1) =\frac{1}{1+c}W(s)+\frac{p}{1+c}V(s+1)+\frac{g'(0)}{1+c}Z(t+1). \end{cases} $$

It is easy to see that when $R_{f}<1$ and $R_{s}\leq1$, then, by conclusion (a) of Theorem 1 and conclusion (a) of Theorem 4 and from the first three equation of (25), we know $(X(t),Y(t),Z(t))\rightarrow(0,0,0)$ as $t\rightarrow\infty$ and further from the last three equations of (25), we also have $(U(s),V(s),W(s))\rightarrow(0,0,0)$ as $s\rightarrow\infty $. Therefore, $D_{0}$ is locally asymptotically stable.

When $R_{f}>1$, then, by conclusion (b) of Theorem 1, we see that equilibrium $(0,0,0)$ of the last three equations of (25) is unstable. In addition, when $R_{s}>1$ we also have $R_{f}>1$. Therefore, $D_{0}$ is unstable.

The linearization system of coupled system (3)–(4) at equilibrium $D_{1}$ is

26 $$ \textstyle\begin{cases} X(t+1) =\frac{1}{1+\mu}X(t)-\frac{\beta A}{\mu(1+\mu)}Z(t), \\ Y(t+1) =\frac{1}{1+\mu+\alpha}Y(t)+\frac{\beta A}{\mu(1+\mu +\alpha)}Z(t), \\ Z(t+1) =\frac{\theta\breve{V}}{1+\gamma}Y(t)+\frac{1}{1+\gamma }Z(t), \\ U(s+1) =\frac{1}{1+m+k\breve{V}}U(s)-\frac{k\breve{T}}{1+m+k\breve {V}}W(s), \\ V(s+1) =\frac{1}{1+m+d}V(s)+\frac{k\breve{V}}{1+m+d}U(s+1)+\frac {k\breve{T}}{1+m+d}W(s), \\ W(s+1) =\frac{1}{1+c}W(s)+\frac{p}{1+c}V(s+1)+\frac{g'(0)}{1+c}Z(t+1). \end{cases} $$

It is clear that when $R_{s}\leq1$, by conclusion (a) of Theorem 4, from first three equation of (26), we know $(X(t),Y(t),Z(t))\rightarrow(0,0,0)$ as $t\rightarrow\infty$. By Theorem 2 and from last three equations of (26), we further have $(U(s),V(s),W(s))\rightarrow(0,0,0)$ as $s\rightarrow\infty$. Therefore, $D_{1}$ is locally asymptotically stable. When $R_{s}> 1$, by conclusion (b) of Theorem 4, we see that equilibrium $(0,0,0)$ of the first three equations of (26) is unstable. Therefore, $D_{1}$ is unstable. This completes the proof. □

However, to establish the criteria of stability for endemic equilibrium $D^{*}$ and two positive equilibria $D_{2}$ and $D_{3}$ is very difficult. We here only give the following conjectures.

### Conjecture 2

*Assume the condition* (A) *holds and one of conditions* (a) *and* (b) *of Lemma *7 *holds*. *Then endemic equilibrium* $D^{*}$ *of coupled system* (3)*–*(4) *is locally asymptotically stable*.

### Conjecture 3

*Assume that* $R_{s}<1$ *and* $H_{M}>0$. *Then* $D_{3}$ *is locally asymptotically stable and* $D_{2}$ *is unstable*.

In the following section, we will give a numerical example to show that Conjectures 2 and 3 may be right.

## Numerical examples

In this section, we give the numerical examples to discuss assumption (A) and the stability for two endemic equilibria $P_{1}$ and $P_{2}$ of slow system (7) and for two equilibria $D_{2}$ and $D_{3}$ of coupled systems (3)–(4). We assume that $s=Kt$ and $t=[\frac{s}{K}]$ in coupled systems (3)–(4). For convenience, we choose $K=366$ and function $g(E)=wE$. The parasite reproduction rate of infected cell *p* is chosen as a free parameter. The rest of the parameters in coupled systems (3)–(4) are chosen in Table 1.

**Table 1:** List of parameters

Parameter	Definition	Value	Source
*A*	the recruitment rate of individuals	4	Ref. [5]
*β*	the infection rate of hosts in a contamination	0.0006	Ref. [5]
*μ*	the natural mortality rate of host	0.0004	Ref. [5]
*α*	the induced mortality rate of host	0.0004	Ref. [5]
*g*(*E*)	the rate which an average host is inoculated	*g*(*E*)=4 × 10^5^*E*	Refs. [5, 6, 23]
*θ*	the rate of contamination	1.5 × 10^−10^	Ref. [5]
*γ*	clearance rate	0.015	Ref. [5]
Λ	the recruitment rate of cells	6000	Ref. [5]
*k*	infections rate of cells	1.5 × 10^−6^	Ref. [5]
*m*	the natural mortality rate of cells	0.3	Ref. [5]
*d*	the induced mortality rate of cells	0.15	Ref. [5]
*c*	the within-host mortality rate of parasites	60	Ref. [5]

By calculating, we obtain

$$(\varphi+1) (n+1) (w-1)M+(1+2r+\varphi)N=0.000026>0. $$

Therefore, assumption (A) is satisfied.

**Figure 1:**
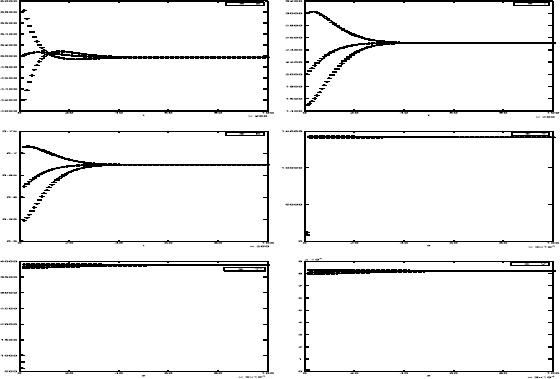
The trajectories of solutions $(S(t),I(t),E(t),T(s),T^{*}(s),V(s))$ with initial values $(S(0),I(0),E(0),T(0),T^{*}(0), V(0))=(4000,1500,0.5,800,800,800), (5000,2000,0.7,1210,1010,1000)$ and $(6000,3000,0.6,850,600,900)$

We first take the parasite reproduction rate of infected cell $p=1200$. By calculating, we see that the basic reproduction numbers $R_{f}\doteq 1.33>1$ and $R_{s}\doteq5>1$. We see that system (7) has only endemic equilibrium $P^{*}(4974.47,2512.76,0.6735)$ and coupled systems (3)–(4) has endemic equilibrium $D^{*}(4974.47,2512.76,0.6735,14\text{,}179.6,3880.26, 82\text{,}095.35)$. The numerical simulations given in Fig. 1 show that equilibrium $P^{*}$ and $D^{*}$ is locally asymptotically stable only when $R_{s}>1$.

**Figure 2:**
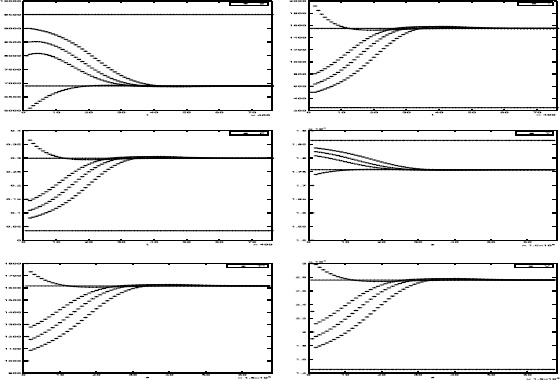
The trajectories of solutions $(S(t),I(t),E(t),T(s),T^{*}(s),V(s))$ with initial values $(S(0),I(0),E(0),T(0),T^{*}(0), V(0))=(8500,600,0.15,15\text{,}000,1000,8500),(8000,800,0.1,16\text{,}000,1200,23\text{,}000)$, $(6000,2000,0.4,19\text{,}000,1800, 30\text{,}000)$ and $(9000,500,0.05,17\text{,}000,1300,19\text{,}000)$

We next take the parasite reproduction rate of infected cell $p=950$. By calculating, we see that the basic reproduction numbers $R_{f}\doteq 1.0556>1$ and $R_{s}\doteq0.8333<1$. Furthermore, we also have $H_{M}=1891.39>0$. Hence, slow system (7) has two endemic equilibria $P_{1}(9503.94,248.02,0.0348)$ and $P_{2}(6900.24,1549.87,0.2995)$ and coupled systems (3)–(4) have two endemic equilibria $D_{2}(9503.94,248.02,0.0348,18\text{,}644.97,903.35, 14\text{,}535.01)$ and $D_{3}(6900.24,1549.87,0.2995,17\text{,}575.93,1616.04,27\text{,}583.87)$. The numerical simulations given in Fig. 2 show that equilibria $P_{2}$ and $D_{3}$ are locally asymptotically stable and equilibria $P_{1}$ and $D_{2}$ are unstable. Therefore, Conjecture 1, Conjecture 2 and Conjecture 3 may be right.

## Discussions

In this paper we studied a discrete coupled within-host and between-host models (3)–(4) in environmentally driven infectious disease obeying Micken’s non-standard finite difference scheme. Since there are two fast and slow time scales in the model, and the fast time scale is sufficiently quicker than the slow time scale, the model is separated into a fast system (6) and a slow system (7).

The basic properties for fast system (6), including the existence of infection-free equilibrium $B_{0}$, infected equilibrium $B^{*}$ (when $E=0$) and infected equilibrium $B_{1}$ (when $E>0$), the positivity and ultimate boundedness of the solutions with positive initial values, are established. Under assumption (H), the local stability of equilibria for system (6) is completely determined by basic reproduction number $R_{f}$. That is, when $E=0$ in system (6), if $R_{f}< 1$ then $B_{0}$ is locally asymptotically stable, and if $R_{f}>1$ then $B_{0}$ is unstable and $B^{*}$ is locally asymptotically stable. When $E>0$ in system (6), then infectious equilibrium $B_{1}$ exists always and also is locally asymptotically stable.

For slow system (7), the basic properties on the existence of disease-free equilibrium $P_{0}$, unique endemic equilibrium $P^{*}$ and two positive equilibria $P_{1}$ and $P_{2}$, and the positivity and ultimate boundedness of the solutions with positive initial values are established.

The sufficient conditions on the local stability of disease-free equilibria $P_{0}$ and unique endemic equilibria $P^{*}$ are established by virtue of basic reproduction number $R_{s}$, the quantity $H_{M}$ and condition (A). However, it is very difficult to discuss the local stability of two endemic equilibria $P_{1}$ and $P_{2}$. Here we only show the local stability of $P_{1}$ and $P_{2}$ by the numerical examples in Sect. 5.

We see that assumption (A) is a pure mathematical condition. It is only used in the proofs of theorems on the local stability of endemic equilibria $P^{*}$ to obtain $|b_{0}|+b_{2}>0$ (see the proof of Theorem 5). Generally, we expect that the local stability of equilibria of slow system (7) can be determined only by basic reproduction number $R_{s}$. Therefore, an open problem is whether condition (A) can be thrown off in Theorem 5. Furthermore, we also do not obtain the global asymptotic stability of equilibria for system (7). The reason is that the construction of Lyapunov function is very difficult.

For whole coupled systems (3)–(4), the basic properties on the existence of infection- and disease-free equilibrium $D_{0}$, viral infection and disease-free equilibrium $D_{1}$, unique endemic equilibrium $D^{*}$ and two endemic equilibria $D_{2}$ and $D_{3}$, and the local stability of equilibria $D_{0}$ and $D_{1}$ are established, respectively. However, it is difficult to discuss the local stability for unique endemic equilibrium $D^{*}$, and two endemic equilibria $D_{2}$ and $D_{3}$. Here, we only show the local stability of $D^{*}$, $D_{2}$ and $D_{3}$ by the numerical examples in Sect. 5.

Comparing the results established in this paper with the results obtained in [1, 3], we see that the dynamical properties of equilibria for discrete-time model (3)–(4) and continuous-time model (2) (see Theorems 1–3 in [1]) in fast time and slow time subsystems, respectively, are very oncoming. This shows that discrete-time model (3)–(4), as a discrete-time analog of continuous-time model (2), is fairly appropriate. Particularly, we can use model (3)–(4) to calculate the numerical approximative solution of model (2) in a neighborhood of equilibrium. In addition, in this paper we further investigate the dynamical properties for whole coupled systems (3)–(4), such as the existence of equilibrium and the local stability of equilibrium.
